# Novel *GUCY2D* mutation causes phenotypic variability of Leber congenital amaurosis in a large kindred

**DOI:** 10.1186/s12881-016-0314-2

**Published:** 2016-07-30

**Authors:** Libe Gradstein, Jenny Zolotushko, Yuri V. Sergeev, Itay Lavy, Ginat Narkis, Yonatan Perez, Sarah Guigui, Dror Sharon, Eyal Banin, Eyal Walter, Tova Lifshitz, Ohad S. Birk

**Affiliations:** 1 Department of Ophthalmology, Soroka Medical Center and Clalit Health Services, Faculty of Health Sciences, Ben Gurion University, Beer Sheva, 84101 Israel; 2 The Morris Kahn Laboratory of Human Genetics, National Institute for Biotechnology in the Negev and Faculty of Health Sciences, Ben Gurion University of the Negev, Beer-Sheva, 84105 Israel; 3 National Eye Institute, National Institutes of Health, Bethesda, MD, USA; 4 Department of Ophthalmology, Hadassah-Hebrew University Medical Center, Jerusalem, Israel; 5 Genetics Institute, Soroka Medical Center, Faculty of Health Sciences, Ben-Gurion University of the Negev, Beer-Sheva, 84101 Israel

**Keywords:** Blindness, Guanylate cyclase, *GUCY2D*, Leber Congenital Amaurosis

## Abstract

**Background:**

Leber congenital amaurosis (LCA) is a severe retinal degenerative disease that manifests as blindness or poor vision in infancy. The purpose of this study was to clinically characterize and identify the cause of disease in a large inbred Bedouin Israeli tribe with LCA.

**Methods:**

Thirty individuals of a single kindred, including eight affected with LCA, were recruited for this study. Patients’ clinical data and electroretinography (ERG) findings were collected. Molecular analysis included homozygosity mapping with polymorphic markers and Sanger sequencing of candidate genes.

**Results:**

Of the eight affected individuals of the kindred, nystagmus was documented in five subjects and keratoconus in three. Cataract was found in 5 of 16 eyes. Photopic and scotopic ERG performed in 5 patients were extinguished. All affected subjects were nearly blind, their visual acuity ranged between finger counting and uncertain light perception. Assuming autosomal recessive heredity of a founder mutation, studies using polymorphic markers excluded homozygosity of affected individuals at the genomic loci of all previously known genes associated with LCA, except *GUCY2D*. Sequencing of *GUCY2D* identified a novel missense mutation (c.2129C>T; p.Ala710Val) resulting in substitution of alanine by valine at position 710 within the protein kinase domain of the retina-specific enzyme guanylate cyclase 1 (GC1) encoded by *GUCY2D*. Molecular modeling implied that the mutation changes the conformation of the regulatory segment within the kinase styk-domain of GC1 and causes loss of its helical structure, likely inhibiting phosphorylation of threonine residue within this segment, which is needed to activate the catalytic domain of the protein.

**Conclusions:**

This is the first documentation of the p.Ala710Val mutation in GC1 and the second ever described mutation in its protein kinase domain. Our findings enlarge the scope of genetic variability of LCA, highlight the phenotypic heterogeneity found amongst individuals harboring an identical LCA mutation, and possibly provide hope for gene therapy in patients with this congenital blinding disease. As the Bedouin kindred studied originates from Saudi Arabia, the mutation found might be an ancient founder mutation in that large community.

## Background

Leber congenital amaurosis (LCA) is a severe inherited retinal dystrophy. It is characterized by blindness or severe visual impairment at birth or within months following birth [1]. The ophthalmological signs diagnostic of LCA are: onset of blindness by the age of one year, sluggish pupillary response, nystagmus, oculo-digital sign, and dramatically reduced or absent electroretinogram (ERG) amplitudes [1]. The incidence of LCA in the general population is between 1/30,000 and 1/81,000 [1]. LCA represents at least 5% of all inherited retinopathies, but this percentage is significantly higher in consanguineous communities [2]. To date, at least 19 genes mutated in patients with LCA have been identified. Most of these mutations are transmitted in autosomal recessive heredity [1].

The purpose of this study was to clinically characterize and identify the cause of disease in a large inbred Bedouin family with multiple members affected by LCA.

## Methods

Thirty individuals of a single Bedouin kindred of the Negev region of southern Israel, including eight affected with LCA, were recruited for this study. Clinical data, including visual acuity, eye movements, anterior and posterior ocular segment findings, as well as ERG results, were collected for the LCA patients.

DNA samples were obtained from affected individuals and their families following approval of the Soroka Medical Center institutional review board and informed consent. Homozygosity at the loci of known LCA genes was assayed for all available DNA samples using polymorphic markers derived from Marshfield maps and novel markers that were designed based on Tandem Repeats Finder (TRF) software and the UCSC Human Genome Database. Intronic primer pairs were designed with the Primer3 (version 0.4.0) software (Whitehead Institute for Biomedical Research, Cambridge, MA), based on DNA sequences obtained from UCSC Genome Browser (sequences available on request). PCR products were separated on polyacrylamide gel using silver staining for detection as previously described [3]. Microsatellite markers used are available upon request. Screening for the mutation of 150 DNA samples of unrelated, ethnically matched control individuals was done by DHPLC as previously described [4].

Multipoint LOD score for the pedigree at the shared locus was calculated using SUPERLINK ONLINE SNP 1.1 (http://cbl-hap.cs.technion.ac.il/superlink-snp/index.php), assuming an autosomal recessive mode of inheritance with penetrance of 0.99 and disease mutant gene frequency of 0.01.

Molecular modeling of protein structure was performed to assess a functional role of a found genetic mutation.

## Results

**Fig. 1:**
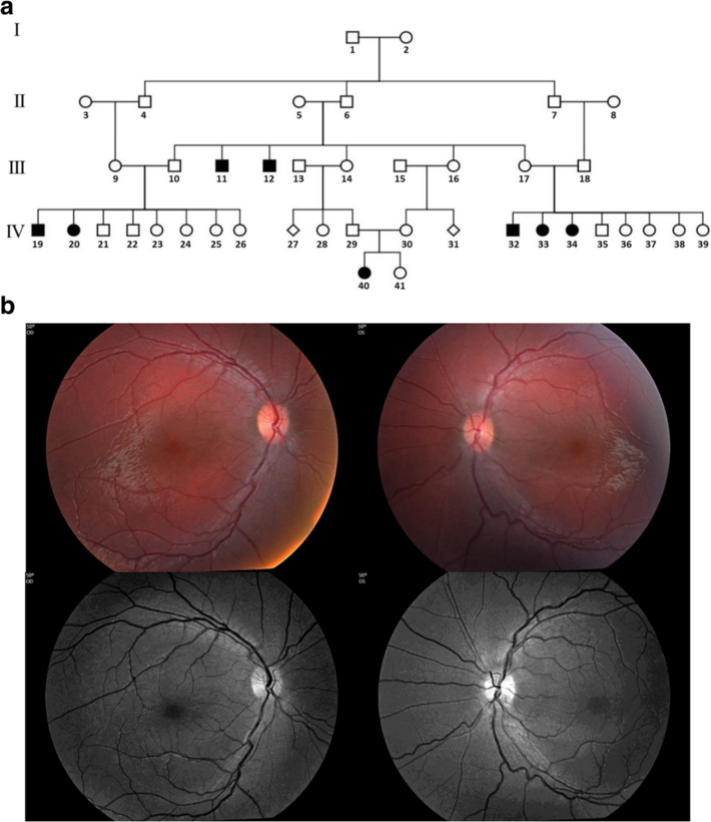
**a** Israeli-Bedouin kindred affected with LCA. Solid and open symbols represent affected and unaffected individuals, respectively. **b** Color (*top*) and red free (*bottom*) fundus photographs of patient 40. Left panels – right eye. Right panels– left eye. Please note normal fundus appearance in both eyes (except for mild vessel tortuosity) in the presence of very poor vision and extinct ERG response

The pedigree was compatible with autosomal recessive heredity due to a founder effect (Fig. 1). All affected subjects were offspring of consanguineous marriages. In all affected subjects, symptoms of poor vision and nyctalopia appeared before one year of age; however, no longitudinal phenotypic data are available. None of them had systemic abnormalities or dysmorphic features. The affected subjects showed visual acuity between finger counting at 0.2 m and uncertain light perception in both eyes (Table 1). Formed nystagmus was documented in five of the eight subjects. The other three had searching eye movements. Three patients had bilateral keratoconus. Cataract appeared in 5 of 16 eyes. Fundus examination was unremarkable in three patients. In the other patients reduced foveal reflex (2 patients), pigmentary changes (2 patients), and changes in retinal vasculature (one patient) were observed (Table 1). ERG was done in 5 patients and all had an extinct response. Figure 1b shows normal fundus appearance of Patient 40, in spite of her severely reduced vision and extinguished ERG. Poor fixation ability, photophobia and media opacities limited clinical data collection and precluded obtaining sufficient quality fundus photographs of additional patients.

**Table 1:** Clinical data of the affected individuals

Pt #	Age (y)	Visual acuity*		ERG	Fundus findings*		Nystagmus		Keratoconus*		Cataract*	
33	11	LP	LP	Extinct	Reduced foveal reflex	Reduced foveal reflex	Yes		No	No	Yes	Yes
34	9	FC 0.15 m	FC 0.2 m	Extinct	Pigmentary changes	Pigmentary changes	Yes		No	No	No	Yes
32	4	ULP	ULP	NA	Reduced foveal reflex	Reduced foveal reflex	No		No	No	No	No
40	3	ULP	ULP	Extinct	Normal	Normal	Yes		Yes	Yes	No	No
11	44	HM	HM	NA	Normal	Normal	No		Yes	Yes	Yes	Yes
12	40	ULP	ULP	Extinct	Normal	Normal	Yes		Yes	Yes	No	No
20	10.5	ULP	ULP	Extinct	Pale, ghost vessels	Pale, ghost vessels	Yes		No	No	No	No
19	12	LP	LP	NA	Pigmentary changes	Pigmentary changes	No		No	No	No	No

Abbreviations: *Pt* patient, *ERG* electroretinogram, *LP* light perception, *FC* finger counting, *ULP* uncertain light perception, *HM* hand motion, *NA* not applicable *Left column shows findings in the right eye, right column – in the left eye

**Fig. 2:**
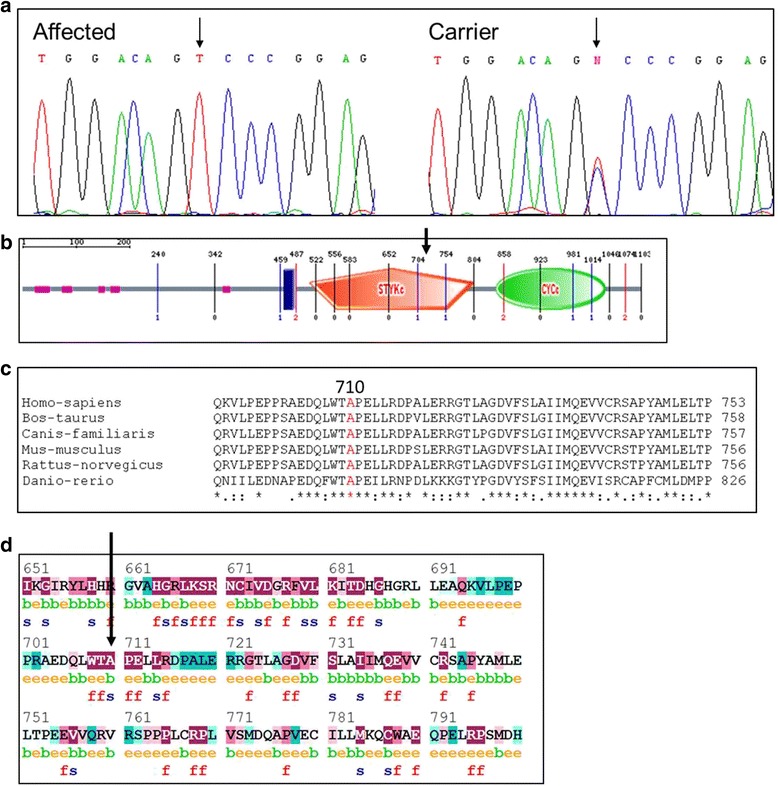
The novel GUCY2D mutation: **a**The c.2129C>T (p.Ala710Val) mutation in *GUCY2D*. Sanger sequence analysis of an affected individual and an obligate carrier. **b** Schematic illustration of the GC1 protein. The mutation occurs within the catalytic protein kinase domain. **c** The alanine at position 710 is highly conserved throughout evolution. **d** ConSeq analysis. Prediction of the structural and functional importance of alanine residue at position 710 was done using the ConSeq server. The ConSeq conservation grade was 9 (meaning highly conserved). Each amino acid is scored on a scale of 1–9, where 1–2, 3–5 and 6–9 signify that the protein sequence is variable, average and conserved, respectively. Predicted functional and structural residues are marked “f” or “s”, respectively. Predicted exposed and buried residues are marked “e” or “b”, respectively

Homozygosity common to all affected individuals was not found in any of the genomic loci of genes previously shown to be associated with LCA, except *GUCY2D,* at 17p13.1 (data not shown). Multipoint LOD score analysis yielded a maximum LOD score of 2.65 at D17S1796. Whole exome sequencing ruled out pathogenic mutations in LCA genes other than *GUCY2D*. Sequencing of the entire coding region of *GUCY2D* and its exon-intron boundaries identified a novel missense mutation (c.2129C>T) in exon 11, resulting in substitution of alanine by valine at position 710 of guanylate cyclase 1 (GC1), the retina-specific enzyme encoded by *GUCY2D* (Fig. 2a). The mutation was not found in any of 150 DNA samples of matched control Bedouin individuals.

**Fig. 3:**
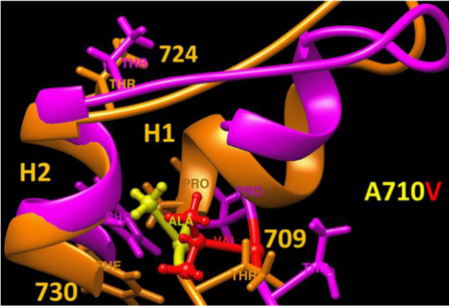
p.Ala710Val mutation causes structural changes in regulatory segment of the kinase styk-domain. The alanine residue is located in the position 710 of a regulatory segment known as a GS region, an approximately 30 amino acids motif that resides within the kinase styk-domain, which has threonine residue accessible for phosphorylation in order to activate the catalytic domain. The fragment of structural superposition of the styk-domains of GUCY2d protein and the p.Ala710Val mutant variant are shown by orange and magenta, respectively. The structure of the styk-domain of the p.Ala710Val mutant variant was equilibrated in water during 30 ns molecular dynamics at 37° to show structural changes due to the mutation. Alanine (*yellow*) is replaced with valine (*red*) residue in the mutant variant p.Ala710Val. The GS region is shown by 2 helices, H1 and H2, connected by a short loop. The p.Ala710Val mutation changes overall conformation of the GS region and causes a partial loss of helical structure in the H1 helix affecting the threonine 709 phosphorylation

The result of molecular modeling of the mutated protein is depicted in Fig. 3. The refined structure of the mutant variant shows that the p.Ala710Val mutation changes the conformation of the regulatory segment within the kinase styk-domain of GC1 and causes loss of its helical structure. These changes are likely to inhibit the phosphorylation of threonine residue within this segment, which is needed to activate the catalytic domain of the protein and thus might affect phosphoregulation of this enzyme [5].

## Discussion

*GUCY2D* (LCA1), localized to 17p13.1, was the first gene described in association with LCA and its mutations constitute the most common cause of the disease, estimated to account for 20 % of LCA cases [6]. The identification of a *GUCY2D* mutation in our Bedouin family is in agreement with the observation that nearly 70% of families with LCA- causing *GUCY2D* mutations originate from Mediterranean countries [7]. The GC1 protein encoded by *GUCY2D* is one of the key enzymes in the photo-transduction cascade. It is located in the disc membranes of both rod and cone photoreceptor outer segments and regulates cGMP and Ca2+ levels within these cells [8]. This enzyme replenishes intracellular cGMP stores, thereby allowing the reopening of cGMP-gated cation channels and the recovery of the depolarized state after excitation of the receptors by light stimulation [9]. Mutations that inactivate GC1 lead to lack of cGMP, persistent closure of the cGMP-gated channels, and therefore a state equivalent to chronic light exposure [6].To the best of our knowledge, this is the first documentation of the c.2129C>T mutation in *GUCY2D*, which causes substitution of alanine by valine at amino acid 710 (Fig. 2b). The mutated alanine at position 710 is highly conserved, suggesting its importance (Fig. 2c). It is predicted to be a structurally significant residue and is within the catalytic protein kinase domain that is essential for GC1 function (Fig. 2d). Molecular modeling of the mutated protein showed alterations in the conformation of the GS region of a catalytic styk-domain (Fig. 3). These changes might affect the proper phosphoregulation involving this enzyme and thus could impair its function [5]. Only one additional mutation within the protein kinase domain of GC1 has been previously described: Perrault et al. found a p.Phe565Ser mutation in consanguineous Arab-Algerian families. The substitution of phenylalanine by serine within the protein kinase domain putatively markedly altered the hydrophobicity of the protein and was expected to affect its stability [9]. Similar to our patients, those described by Perrault et al. with p.Phe565Ser and other *GUCY2D* mutations, expressed inability to follow light or objects, had roving eye movements, nystagmus, severe photophobia and non-recordable ERG [9].

Genetic heterogeneity of LCA is well known and genotype–phenotype correlations with gene-specific phenotypic features have been established, where LCA1 has been typically associated with non-evolutive congenital blindness [1, 9]. However, phenotypic variability among LCA patients who harbor the same mutation in the same gene has not been thoroughly reported and discussed. The large kindred studied enabled a unique opportunity for clinical analysis of phenotypic heterogeneity of individuals with the same mutation. It is of interest to note that while all affected individuals studied have an identical homozygous *GUCY2D* mutation, variation was seen in the clinical phenotype, highlighting the scope of phenotypic heterogeneity in LCA, even within affected families: while all affected individuals had poor vision and all recorded ERG responses were extinct, the specific phenotypes differed: nystagmus was documented in only five patients, keratoconus in three, and cataract was found in 5 of 16 eyes.

The identification of a new *GUCY2D* mutation in this large kindred enlarges the scope of genetic variability and enables pre-natal diagnosis for LCA in the large tribe studied. It also provides hope for treatment for patients with this severe debilitating disease, as gene therapies for LCA are rapidly evolving: recent human trials with gene augmentation therapy for LCA2 caused by mutations in *RPE65* demonstrated lasting improvements in retinal and visual function following the administration of the vector carrying the gene [10–13]. Pasadhika et al. have shown that LCA1 patients retain all retinal layers, and might benefit from gene therapy even more than other forms of LCA, where photoreceptor cell survival is compromised earlier [14, 15]. Studies of GC1-deficient mice showed restoration of rod and cone function and an improvement in visual behavior following subretinal delivery of the gene by a viral vector, adeno-associated virus [16, 17]. These results, which lasted at least 6–11 months, provide firm basis for human therapeutic trial for LCA1 which is currently underway [18].

## Conclusions

We report phenotypic variability among members of a large Bedouin-Israeli family affected by LCA1 due to homozygosity of a novel *GUCY2D* mutation. This mutation, c.2129C>T, causes substitution of alanine by valine at amino acid 710 in the *GUCY2D*-encoded retina-specific enzyme, GC1. The mutated alanine is highly conserved and is predicted to be a structurally significant residue; it is located within the catalytic protein kinase domain that is essential for GC1 function. Only one additional mutation within the protein kinase domain of GC1 has been previously described. Molecular modeling of the mutated protein showed structural changes within the protein that might affect the proper phosphoregulation involving this enzyme and thus could impair its function. Our findings enlarge the scope of genetic variability of LCA and enable pre-natal diagnosis in a large tribe affected with this blinding disease. Moreover, it is of interest that the Bedouin kindred studied originates from Saudi Arabia. Thus, the mutation found might be an ancient founder mutation in that large community.

## Abbreviations

ERG, electroretinography; LCA, Leber congenital amaurosis; TRF, Tandem Repeats Finder
